# Reproductive Physiology, Genetic Architecture, and Management of Duolang Sheep Under Arid-Zone Production Systems: A Review

**DOI:** 10.3390/ijms27104554

**Published:** 2026-05-19

**Authors:** Gul Muhammad Shahbaz, Muhammad Sajid, Huiping Sun, Chenglon He, Lexiao Zhu, Wei Li, Ruohuai Gu, Chaofan Wang, Shuxin Chen, Feng Xing

**Affiliations:** Key Laboratory of Tarim Animal Husbandry Science and Technology, Xinjiang Production and Construction Group, School of Animal Science and Technology, Tarim University, Alar 843300, China; shahbazbalouch42@gmail.com (G.M.S.); msajidkhan663@gmail.com (M.S.);

**Keywords:** Duolang sheep, reproductive efficiency, genomic selection, environmental stress, litter size

## Abstract

Duolang sheep, an indigenous breed of southern Xinjiang, are significant for their agricultural systems due to their adaptation to arid and semi-arid environments. This review integrates recent advancements in Duolang’s reproductive biology, genomic studies, and management strategies to address the breed’s reproductive efficiency under challenging ecological conditions. Reproductive traits such as puberty onset, estrous cycle characteristics, and seasonal breeding are influenced by complex genetic and several environmental factors. Numerous remarkable genomic findings highlight key loci related to fecundity, including the Booroola FecB mutation, as well as genes involved in steroidogenesis, folliculogenesis, and HPG axis regulation. Despite the genetic potential for increased prolificacy, Duolang sheep often exhibit low litter sizes, largely constrained by detrimental environmental factors and management practices. This review emphasizes the significance of integrating genetics, nutrition, and reproductive management to optimize productivity. Strategies such as nutritional flushing, hormone-based estrous synchronization, and selective breeding for increased litter size are discussed, with a focus on minimizing the risks associated with early puberty and lamb survival. Furthermore, the review explores the potential of genomic selection, marker-assisted breeding, and advanced reproductive technologies to enhance the breed’s performance. Finally, the review outlines future research directions, necessitating the development of genomic resources, precise breeding programs, and field trials on reproductive interventions to accelerate genetic gains in Duolang sheep. This integrated approach promises to improve reproductive outcomes, with implications for sustainable sheep production in Xinjiang and similar environments across the globe.

## 1. Introduction

Sheep play a pivotal role in meat production, as a source of revenue, and in sustaining a sustainable livelihood in many dryland regions. Reproductive efficiency is the principal biological determinant of flock profitability. Traits such as age at puberty, lambing interval, litter size, and lamb survival together determine the number of lambs weaned per ewe per year and have a larger economic impact than most growth or carcass traits, particularly in low-input pastoral systems [1,2]. In northwest China, Xinjiang is a foremost producer of sheep despite being characterized by arid and semi-arid climates, low annual rainfall (typically <150–300 mm, concentrated in late spring and early summer), and strong seasonal variations in temperature and forage availability [3,4]. Duolang sheep are native, fat-tailed meat types concentrated in southern Xinjiang, especially in the oasis–desert fringe of Kashgar Prefecture. They are valued for carcass yield, growth performance, and adaptation to harsh rangeland conditions, and recent genomic studies show that Duolang form a distinct genetic cluster [5,6]. Endocrine and transcriptomic work indicates early sexual maturity and relatively weak photoperiod sensitivity in Duolang ewe lambs, while whole-genome and candidate-gene analyses have identified fecundity-related variants, including the Booroola FecB mutation and other loci involved in folliculogenesis and steroidogenesis [7,8]. At the same time, field data suggest that most flocks still have an average litter size close to one, indicating a gap between genetic potential and realized reproductive output under current management [9]. Phenotypically, Duolang sheep are described as early-maturing, good-growth meat animals with favorable reproductive potential [5], and several reports suggest extended or near-year-round estrous activity atypical of many temperate breeds [1]. Recent genomic and transcriptomic work on litter size and ovarian pathways positions Duolang alongside other prolific northwest Chinese breeds while highlighting that realized prolificacy remains strongly conditioned by season, nutrition, and management intensity [6,7]. The production environment of Duolang sheep is described in detail in Section 2 [8].

## 2. Breeding and Production Context in Duolang Sheep

**Figure 1:**
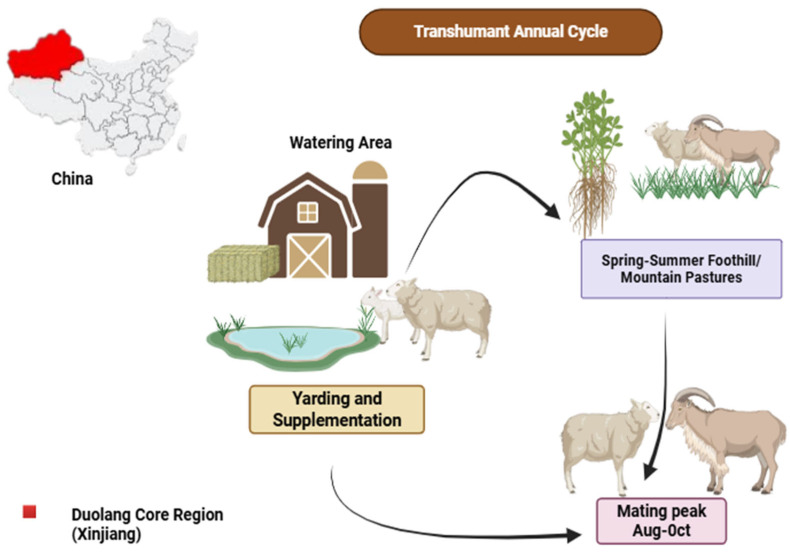
The figure shows the traditional seasonal movement of Duolang sheep between village-based watering and yarding areas and spring–summer foothill or mountain pastures. Supplementation is provided when forage availability declines. The main mating peak occurs in August–October under extensive management. The diagram summarizes the links among seasonal movement, feeding support, and breeding timing under arid-zone conditions.

Duolang sheep are native meat fat type breed from southern Xinjiang, with core populations concentrated in Kashgar Prefecture, especially Maigaiti/Markit County and neighboring oasis counties along the western edge of the Tarim Basin, where they are raised in mixed agro-pastoral systems, grazing desert and foothill rangeland from spring to autumn and receiving yarded or stall supplementation near villages in winter [9]. The management of Duolang sheep in southern Xinjiang follows a distinct transhumant cycle, moving between water zones, summer pastures, and yarding areas (Figure 1). This cycle is heavily influenced by environmental factors, such as temperature extremes and forage availability, which in turn shape reproductive performance (Figure 1). Historical evidence and population-genomic analyses indicate that Duolang sheep originated as a composite breed formed in Xinjiang in the early 20th century and subsequently underwent local adaptation and consolidation as a genetically distinct cluster under hot–arid, desert oasis conditions [10]. Genome-wide SNP and resequencing studies uniformly differentiate Duolang from other Xinjiang breeds (e.g., Altay, Bashbay) and from imported meat breeds (e.g., Suffolk, Dorset), revealing selection signatures related to thermotolerance, energy metabolism, and reproductive function that reflect long-term adaptation to thermal extremes, water and forage scarcity, and extensive management [11,12,13]. Phenotypically, Duolang sheep exhibit several unique characteristics, including primal and efficient growth, and meat animals with favorable reproductive potential. Several reports suggest extended or near-year-round estrous activity, which is atypical of many temperate breeds [14,15]. Recent genomic and transcriptomic work on litter size and ovarian or hypothalamic pathways positions Duolang besides other prolific north-west Chinese breeds while highlighting that realized prolificacy remains strongly conditioned by season, nutrition, and management intensity [16,17]. Because most Duolang flocks are clustered in the Kashgar-area oasis–desert fringe systems, the specific environmental drivers (heat load, aridity, seasonal feed availability) and local husbandry practices in this region provide essential context for interpreting the puberty timing, estrous expression, conception rates, and lamb survival outcomes discussed in subsequent sections.

## 3. Reproductive Physiology Overview

In ewes, reproductive physiology is governed by a well-coordinated neuroendocrine gonadal system that mediates the transition from anestrous to ovarian periodicity, controls the estrous cycle, ensures ovulation, supports luteal physiology, and maintains pregnancy. The process begins in the hypothalamus with pulsatile release of a gonadotropin-releasing hormone (GnRH), which stimulates the anterior pituitary to secrete a follicle-stimulating hormone (FSH) and luteinizing hormone (LH) [18]. FSH promotes early follicular growth and wave-like development of antral follicles, while rising estradiol from dominant follicles exerts positive feedback to trigger the preovulatory LH surge and subsequent ovulation [19,20]. The classic ewe estrous cycle averages about 16–17 days in length during the breeding season (range ≈ 14–19 days), with estrous lasting approximately 24–36 h and ovulation occurring toward the end of estrous or roughly ~24 h after estrous onset [19,21,22]. After ovulation, the corpus luteum forms and secretes progesterone, which inhibits further follicular recruitment and prepares the uterus for possible implantation; if pregnancy does not occur, luteolysis is initiated by pulsatile prostaglandin F2α (PGF2α) from the uterus around day ~14, allowing a new follicular wave to commence [19,20].

Sheep are generally seasonal breeders in temperate latitudes, exhibiting anestrous during spring/summer when day length is long; declining photoperiod after the summer solstice stimulates melatonin secretion from the pineal gland, which modulates GnRH neurons and re-initiates cyclicity [3,5,6]. However, the exact length of anestrous and sensitivity to photoperiod vary by breed, latitude, and management, such that breeds in more arid or subtropical zones may show extended or year-round estrous [21]. At the ovarian level, follicular growth occurs in waves, typically 3–4 waves per interovulatory interval. Each wave is initiated by an FSH rise, followed by the selection of a dominant follicle that secretes estradiol and becomes ovulatory, while subordinate follicles undergo atresia [19]. Luteal progesterone supports early embryonic development, maintains uterine quiescence, modulates immune tolerance, and regulates uterine secretions. In addition to classical steroid feedback, endocannabinoid, kisspeptin, and neuro-opioid pathways are increasingly recognized as important modulators of GnRH pulsatility and seasonal transitions in small ruminants, integrating metabolic and environmental cues (nutrition, body condition, photoperiod, temperature) into central reproductive control [23]. Pregnancy in sheep begins with maternal recognition of pregnancy mediated by conceptus-derived interferon tau (IFNT), typically by day ~12–13 after mating. IFNT acts on the endometrium to inhibit oxytocin receptor upregulation and to suppress pulsatile uterine PGF2α secretion, thereby rescuing the corpus luteum and sustaining progesterone production [24]. As gestation progresses, uterine and placental hormones, including estrogens, prolactin, relaxing, oxytocin, and various growth factors, help to maintain pregnancy, promote fetal development, and eventually trigger parturition. Given this physiological background, reproductive performance metrics (age at first estrous/lambing, estrous cycle length, conception rate, ovulation rate, litter size, lambing interval) are intimately tied to the integrity of the hypothalamic–pituitary–gonadal axis, follicular dynamics, luteal competence, and the ewe’s responses to photoperiod, nutrition, thermal environment, and handling. For the breed-focused sections that follow (Duolang sheep), these physiological principles provide the reference framework for interpreting how breed-specific performance may deviate or adapt under the southern Xinjiang environment [19,21].

## 4. Onset of Puberty and Sexual Maturity in Duolang

Recent neuroendocrine and ovarian studies highlighted that Duolang ewe lambs attain their first estrous at approximately 145–150 days of age (≈4.8–5.0 months), consistent with an early-maturing profile and reports of extended, often year-round, cyclicity in southern Xinjiang breeds adapted to desert–oasis systems [25,26]. Immunohistochemical and expression analyses have further characterized ERα and ERβ dynamics across the hypothalamus, pituitary, and ovary during this transition, implicating ERα in central feedback regulation and ERβ in ovarian maturation [27,28]. Complementary hypothalamic transcriptome studies across pre-pubertal, pubertal, and post-pubertal stages identify lncRNA–mRNA networks and differentially expressed genes related to steroidogenesis, follicle growth, and oocyte competence, supporting a coordinated central-ovarian program that times first ovulation near five months under adequate nutrition and management [25,28]. Although physiological puberty can occur around this age, recommended first mating in extensive southern Xinjiang systems is typically later (often up to ~18 months) to allow attainment of target body weight and to reduce dystocia risk, reflecting growth, seasonality, and feed constraints rather than endocrine immaturity [29]. In more intensified agro-pastoral settings with improved nutrition, body weight thresholds (e.g., ≥65–70% of mature ewe weight) are used to schedule first service soon after puberty, and emerging Duolang genomic resources paired with litter size phenotypes offer the possibility of selecting for earlier, well-supported sexual maturity without compromising lifetime fertility [30].

## 5. Estrous Cycle Characteristics of Duolang Sheep

**Figure 2:**
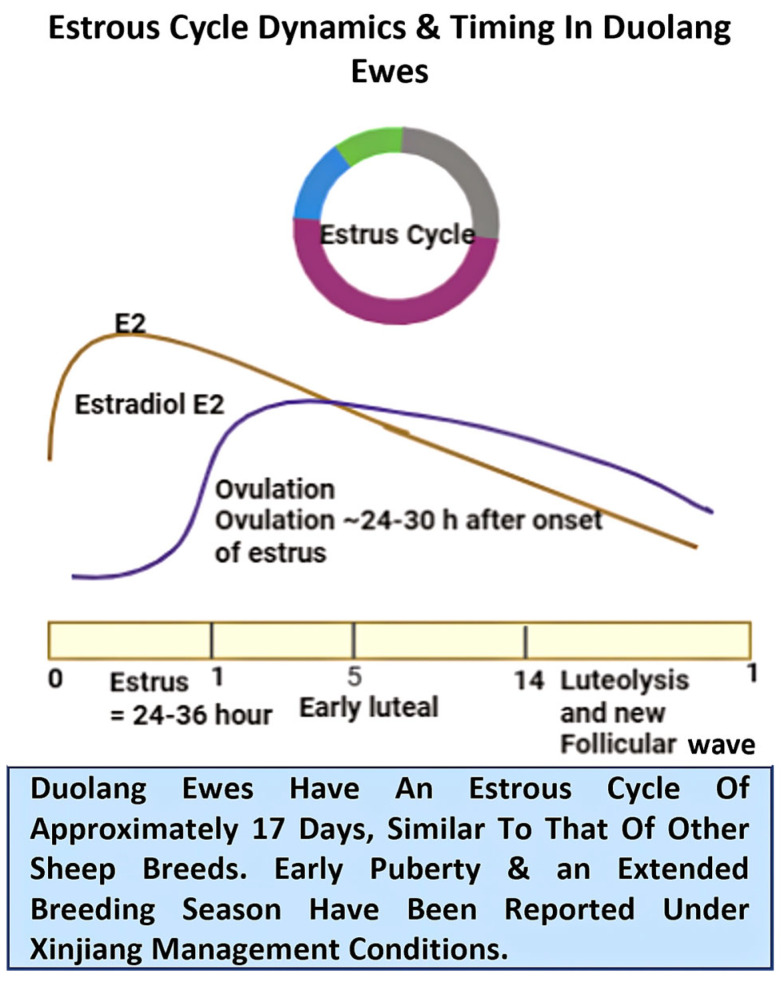
Estrous cycle dynamics and timing in Duolang ewes. The figure summarizes the approximate 17–day estrous cycle reported for Duolang sheep under Xinjiang management conditions, including estrus duration (about 24–36 h), expected timing of ovulation (approximately 24–30 h after onset of estrus), and the transition through the early luteal, luteolysis, and follicular phases. The schematic also indicates the general pattern of estradiol (E2) changes across the cycle and highlights that Duolang ewes appear broadly similar to other sheep breeds, while an extended breeding season has been reported under local production conditions.

In Duolang ewes, available data demonstrate that estrous cycle characteristics mainly follow the classical ovine pattern, with inter-estrous intervals averaging about 17 days (range ≈ 14–20) during the breeding season [31,32,33]. Estrus usually lasts about 24–36 h, and ovulation generally occurs 20–40 h after its onset, near the end of behavioral estrous [32,33]. The timing of estrous, ovulation, and luteal regression in Duolang ewes is summarized in Figure 2. As in other sheep, Duolang are seasonally polyestrous; however, practical observations from oasis–desert systems in southern Xinjiang suggest a relatively extended breeding season compared with strongly seasonal temperate breeds, with estrous expression strongly influenced by nutrition and heat load [34]. Management recommendations for natural service and AI are based on these temporal dynamics: estrous-to-ovulation intervals of about 24–30 h and intravaginal progesterone pessary to ovulation of roughly 48–65 h underpin common fixed-time artificial insemination (FTAI) protocols. In practice, insemination is typically performed 12–18 h after the onset of estrous to optimize fertilization success. Both progesterone- and PGF2α-based synchronization protocols (with or without GnRH) are used to control follicular dynamics and precisely align ovulation with insemination timing [31,35]. Environmental and nutritional modifiers further influence reproductive outcomes. Heat stress can shorten estrous, alter cycle expression, depress estrous behavior, and increase early embryo loss, whereas short-term “flushing” before mating (additional energy/protein) generally increases ovulation rate and fecundity by improving oocyte quality [36,37]. Duolang-focused molecular studies, including ovarian transcriptome analyses and embryo transfer/AI experiments, indicate that the breed responds predictably to synchronization and superovulation; however, high ambient temperatures in southern Xinjiang can compromise embryo quality, emphasizing the importance of appropriate seasonal scheduling of reproductive interventions [38].

## 6. Reproductive Performance Metrics in Duolang Sheep

In Duolang sheep, reproductive performance can be described using a few key indicators: gestation length, inter-lambing interval, litter size, twinning rate, and lamb survival. Core reproductive performance indicators for Duolang sheep under southern Xinjiang production systems are summarized in Table 1 [39,40]. Gestation length in Duolang ewes is similar to that of other meat breeds, ranging from 147 to 152 days, with only minor variations due to fetal number, parity, and late-gestation nutrition; therefore, it is not considered a major constraint [41]. Under traditional extensive grazing systems in southern Xinjiang, mating is aligned with seasonal forage availability, resulting in an effectively annual lambing pattern with approximately one lambing per ewe per year. In principle, improved nutrition, flushing, targeted supplementation, and estrous synchronization could reduce the inter-lambing interval to 8–9 months; however, robust field data from Duolang flocks are still limited [40,42]. Current farm reports indicate that realized litter size is close to 1.0 in most systems, with ewes typically producing a single lamb per lambing, reflecting the breed’s historical role as a fast-growing meat sheep rather than a specialized prolific maternal line [43]. However, genomic and transcriptomic studies show that Duolang carries allelic variation in reproduction-related genes such as WWC2, SLK, ARHGEF9, FSHR, INSL3, and WNT2B, and that the Booroola FecB mutation is present in at least part of the population, indicating that litter size is biologically responsive to marker-assisted and genomic selection if breeders choose to emphasize prolificacy [17,44]. Because most lambings are singletons, perinatal survival in Duolang is generally high, and, as in other well-managed meat flocks, losses are concentrated in the first 24–48 h after birth and mainly relate to dystocia, neonatal hypothermia, poor colostrum intake, and infectious disease; sheltered lambing areas, close supervision, prompt assistance to weak lambs, and micronutrient supplementation in late gestation are expected to maintain high survival and to buffer any increase in mortality if twinning rates are raised [45]. This combination of moderate prolificacy, strong growth performance, and adaptation to arid environments, together with evidence of prolificacy alleles such as FecB, positions Duolang as a potential dual-purpose genetic resource in which modest, controlled gains in litter size can be pursued without compromising meat production or environmental resilience [43,44].

## 7. Seasonality and Out-of-Season Breeding in Duolang Sheep

Duolang sheep are managed in the arid and semi-arid environments of southern Xinjiang, where traditional transhumant calendars and forage dynamics strongly shape the timing of mating and lambing [48,49]. Flocks typically move to higher pastures in late spring–summer and return to winter pastures in late autumn, and, as in other mid-latitude (~35–45° N) systems, most indigenous breeds show a main breeding season in late summer–autumn, with lambing concentrated in late winter or early spring when forage availability begins to improve [50,51]. Field observations from Xinjiang grasslands indicate that mating activity and ram introduction commonly peak from August to October, sometimes supported by controlled mating or AI to tighten lambing patterns, and Duolang flocks appear to follow this general autumn-mating/spring-lambing pattern under extensive management, with greater flexibility in semi-intensive farms [49]. Reproductive seasonality in sheep is primarily regulated by photoperiod via melatonin-mediated control of hypothalamic–pituitary–gonadal activity [52]. However, in Duolang sheep, temperature and forage conditions strongly interact with this framework. Hot, dry summers and poor pasture availability can delay the onset of cyclicity or suppress ovulation, whereas improved autumn nutrition enhances ovulation and conception. Winter conditions may also influence lambing timing by reducing the risk of neonatal losses [53].

**Figure 3:**
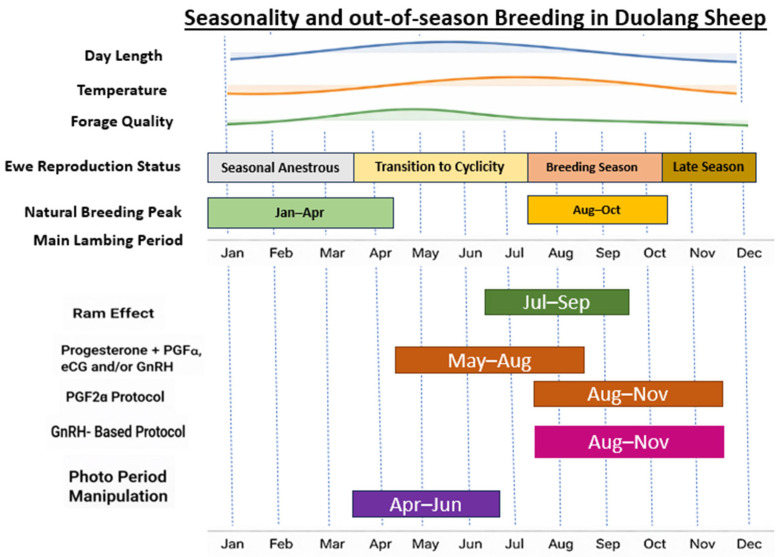
Seasonal reproductive calendar and recommended intervention windows in Duolang sheep under southern Xinjiang conditions. Day length, temperature, and forage quality are shown qualitatively together with ewe reproductive status across the annual cycle. Under extensive management, the natural breeding peak occurs mainly from August to October, whereas the main lambing period occurs mainly from January to April. The lower panel shows the recommended timing of key interventions used to advance, synchronize, or extend the breeding season, including the ram effect, progesterone device + eCG (±PGF_2_α), PGF_2_α protocols, GnRH–based protocols, and photoperiod manipulation. PGF_2_α-only and GnRH–based protocols are not recommended during deep seasonal anestrus, and ram effect is most effective near the onset of the breeding season.

Genomic studies in Xinjiang and other Chinese sheep have identified selection signatures in genes related to reproduction and environmental adaptation, suggesting that Duolang sheep may possess a genetic basis for extended or less strictly seasonal breeding that can be expressed under improved management [54]. Seasonal patterns in day length, temperature, and forage quality, together with the recommended windows for key interventions used to advance or extend the breeding season in Duolang sheep, are illustrated in Figure 3, whereas the main protocols and their practical interpretation are summarized in Table 2. Several interventions are used to advance or extend the breeding season in Duolang flocks. Artificial photoperiod manipulation (controlled light exposure), the ram effect, and hormonal synchronization protocols (progesterone devices with PGF2α, eCG, and/or GnRH) are commonly applied to induce and synchronize estrous and facilitate fixed-time AI [55,56,57,58]. While effective, these approaches require appropriate management inputs, including nutrition, labor, and infrastructure, to avoid negative impacts on ewe condition and welfare [51]. In practice, combining moderate hormonal use with the ram effect and improved nutritional management provides a more sustainable strategy for extending the breeding season and enhancing reproductive efficiency in Duolang sheep under arid production systems.

## 8. Management Interventions

Effective reproductive management is essential for translating the biological potential of Duolang sheep into improved flock productivity, particularly in the arid and semi-arid systems of southern Xinjiang. As a meat-type breed often exposed to fluctuating forage and climatic stress, Duolang ewes benefit strongly from targeted nutritional strategies before mating and around lambing. Nutritional flushing, short-term supplementation of energy and protein 2–4 weeks pre-mating, has been shown to increase ovulation rate, follicular growth, and conception in ewes by elevating metabolic signals such as glucose, insulin, IGF-1, and leptin, which support follicular development [67]. In Xinjiang pastoral systems, where summer forage quality typically declines, flushing and maintaining ewes at a body condition score (BCS) of about 2.75–3.25 at mating are particularly important for maximizing conception and reducing early embryonic loss [68]. In late gestation, when ≈70% of fetal growth occurs, inadequate energy or protein intake increases the risk of low birth weight, weak lambs, and perinatal mortality; supplementation with rumen-bypass protein, vitamin E and selenium improves colostrum yield, oxidative status and lamb survival, which is especially relevant for Duolang ewes lambing in late winter or early spring when pasture is scarce [59,69].

Beyond nutrition, a range of endocrine and management interventions can be used to control estrous and lambing patterns in Duolang flocks. Progesterone-based protocols using intravaginal devices (sponges or CIDR inserts) remain the main method for estrous synchronization and fixed-time AI; devices are typically left in place for 12–14 days, and at device removal, ewes commonly receive eCG, with PGF2α added in some protocols depending on cyclic status and the presence of functional corpora lutea. [61]. This combination induces a synchronized estrous 24–72 h after withdrawal and supports predictable FTAI with acceptable pregnancy rates, allowing Duolang producers to concentrate lambing and generate off-season lamb crops where hygiene, nutrition, and costs are properly managed [62,63]. PGF2α-based protocols are limited to cycling ewes; a common double-injection regimen given 9–11 days apart produces good estrous synchrony and fertility at low cost and without intravaginal devices, making it well-suited to natural-mating Duolang flocks with limited infrastructure [64]. GnRH-based, Ovsynch-type sequences (GnRH–PGF2α–GnRH), often combined with short-term CIDR use, can further refine control of ovulation timing and are promising for semi-intensive Duolang nucleus flocks where FTAI is routine [65]. Non-hormonal bio stimulation via the ram effect, introducing previously isolated, sexually active rams to anestrous ewes, remains a particularly attractive option for low-input systems, inducing ovulation within 48–72 h and tightening mating and lambing periods, especially during seasonal transition periods and when ewes have BCS ≥ 2.5 [59,60]. The ram effect can be further potentiated by brief progesterone priming or low-dose PGF2α/eCG to improve synchrony and conception [61,64]. Finally, controlled photoperiod manipulation, in which rams or ewes are exposed to artificial long days (≈16 h light/day for 6–8 weeks) followed by naturally decreasing day length, can be used to advance or extend the breeding season. Although light control systems are feasible mainly in semi-intensive farms, they may improve semen quality and ram libido during spring and summer and may be particularly useful in elite Duolang nucleus flocks [66]. Together, these nutritional, hormonal, bio stimulatory, and photoperiodic tools provide a flexible management toolbox, but their use must be balanced against costs, labor, welfare, and the nutritional capacity of Duolang ewes to sustain higher reproductive output under Xinjiang conditions.

## 9. Genetics and Omics of Reproduction in Duolang Sheep

**Figure 4:**
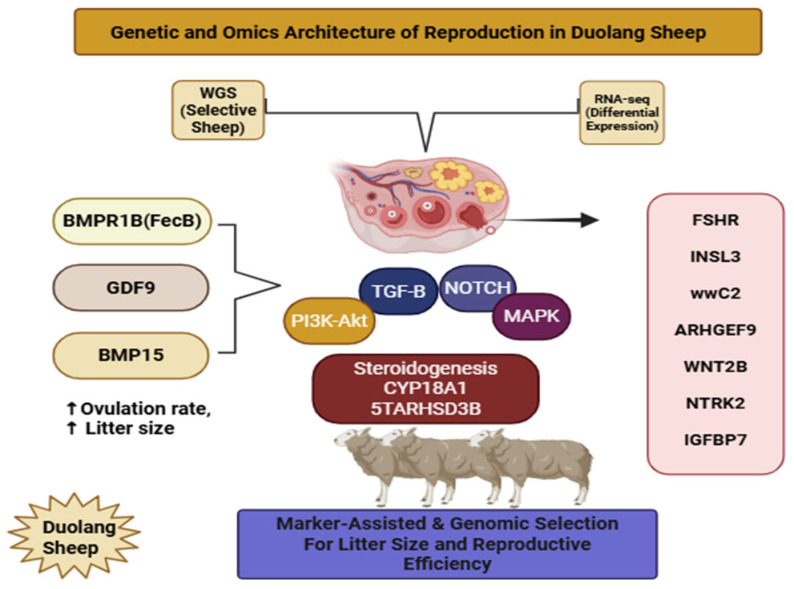
Genetic and omics factors affecting reproduction in Duolang sheep. The figure highlights major fecundity genes linked to ovulation rate and litter size. It also summarizes candidate genes identified by WGS and RNA–seq. Key pathways involved in ovarian function and steroidogenesis are shown. These markers may support genomic selection for improved reproductive efficiency.

Reproductive traits in sheep are polygenic, with low to moderate heritabilities and strong modulation by nutrition, season, and management [70]. In Duolang and other Chinese indigenous breeds, candidate-gene studies and whole-genome analyses indicate that fecundity variation arises from the joint action of major TGF-β fecundity genes, endocrine receptors, and numerous small-effect loci involved in folliculogenesis, steroidogenesis, and hypothalamic–pituitary–ovarian (HPO) signaling [71]. Central among these are the major fecundity genes BMPR1B (FecB), GDF9, and BMP15, which together form the core of the classical ovine fecundity-gene network, now complemented by newer multi-breed SNP surveys of BMP15 variation [72]. The main candidate genes, ovarian signaling pathways, and omics tools identified in Duolang sheep are summarized in Figure 4. The Booroola FecB mutation increases ovulation rate and litter size by approximately one to two lambs per lambing in heterozygous and homozygous carriers, whereas GDF9 and BMP15 mutations alter oocyte–granulosa communication and follicle survival, with heterozygotes showing increased ovulation and homozygotes often becoming infertile due to arrested folliculogenesis [73]. In prolific breeds, combinations of BMPR1B, BMP15, and FSHR polymorphisms are significantly associated with increased litter size, with multi-locus genotypes displaying additive or synergistic effects on ovulation rate and litter size [74]. Work in Xinjiang has confirmed BMPR1B as a key candidate for prolificacy in Duolang, with FecB-carrying ewes exhibiting superior litter size compared with non-carriers, and more recent studies have implicated additional loci such as INSL3, WNT2B, and other ovarian-pathway genes in variation in ovulation rate and litter size in local breeds, indicating a more complex network beyond the classical FecB/GDF9/BMP15 axis [46].

High-density SNP chips and whole-genome resequencing (WGS) have further revealed selection signatures for reproduction in north-western Chinese sheep: in particular, sweeps harboring PAK1, CYP19A1, PER1, and other genes involved in ovarian steroidogenesis, circadian regulation, and seasonal reproduction distinguish Duolang from high-prolific Hu sheep, while broader resequencing work shows that many reproduction-related regions overlap with loci under adaptation to altitude and aridity, supporting a tight coupling between reproductive timing and environmental stress [71]. RNA-seq and multi-tissue transcriptome studies in prolific Chinese lines demonstrate that reproductive performance reflects coordinated expression changes across the HPO axis, with enrichment of NOTCH, MAPK, PI3K–Akt and steroidogenesis pathways (e.g., CYP19A1, STAR, HSD3B) in ovary, uterus and hypothalamus; high-fecundity ewes consistently show upregulation of pro-growth and anti-apoptotic genes and downregulation of inhibitory signaling, indicating that differences in litter size arise partly from altered follicle recruitment and atresia rather than solely from terminal ovulatory events [73]. Although Duolang-specific ovarian transcriptomes remain limited, existing WGS and candidate-gene results suggest that Duolang shares many of these regulatory pathways with other prolific Chinese breeds, but with distinct allele frequencies and selection histories reflecting its adaptation to arid Xinjiang. Meta-analyses across breeds show that heritability for litter size is low (h2 ≈ 0.05–0.15), whereas ovulation rate, age at puberty, and growth traits typically show higher heritabilities, implying that traditional phenotypic selection for litter size alone will be slow. By contrast, whole-genome information through marker-assisted selection using validated BMPR1B/BMP15/GDF9/FSHR variants and, more powerfully, genomic selection based on dense SNP panels can increase the accuracy of breeding values for low-heritability reproductive traits while controlling inbreeding [75]. For Duolang sheep, integrating WGS-derived selection signatures, reproduction-related candidate genes, and genomic prediction models tailored to Xinjiang production systems would enable the development of Duolang-specific genomic selection indices that jointly weight litter size, lamb survival, and environmental robustness, accelerating reproductive progress beyond what is achievable through conventional selection alone.

## 10. Comparative Context (Chinese and Global Breeds)

Reproductive performance in sheep varies widely across breeds as a consequence of evolutionary history, domestication trajectories, environmental pressures, and deliberate selection for maternal versus meat traits, and this broader context helps to position Duolang sheep within both Chinese and global benchmarks. Comparative litter size at birth for Duolang and selected prolific breeds is summarized in Table 3. Within China, Hu sheep are widely regarded as the premier high-prolificacy model, with mean litter sizes of about 2.0–2.4, exceptionally early puberty and extended or nearly a seasonal estrous activity under controlled feeding; prolificacy in Hu is strongly associated with major fecundity genes such as BMPR1B (FecB), GDF9 and BMP15 and with upregulation of key folliculogenesis and steroidogenic genes (e.g., CYP11A1, IGFBP7, WNT4), setting an upper biological benchmark for litter size and ovulation rate in Chinese breeds [76,77]. Small-Tail Han (STH) sheep show similar or even higher prolificacy, with reported litter sizes around 2.4–2.8 depending on nutrition and parity, and combine high twinning and triplet rates with strong maternal behavior and high lamb survival; genomic analyses in STH highlight selection on ovarian steroidogenesis (e.g., HSD3B, STAR), follicular survival (PI3K–Akt) and cell-cycle regulation pathways, supporting high conception rates in accelerated lambing systems. By contrast, Tan sheep represent a more conservative reproductive strategy, with moderate litter size (≈1.4–1.6) but very high lamb survival and resilience to cold, drought, and forage scarcity, underpinned by the upregulation of oxidative defense and cellular stress genes (e.g., SOD2, HSP70) and strong melatonin responsiveness, effectively prioritizing survival over maximal fecundity [78]. Duolang sheep therefore occupy an intermediate position among Chinese breeds: less prolific than Hu or STH, but with stronger meat performance and environmental robustness, and with genomic evidence (e.g., presence of FecB and other reproduction-related alleles) indicating scope for measured increases in prolificacy under targeted selection. In the global context, breeds such as Romanov and Finnsheep, which exhibit extreme prolificacy, illustrate the upper biological limits of ovulation rate and litter size. Romanov sheep often produce 2.7–3.0 lambs per lambing and lamb crops of 300–350% under good management, supported by weak seasonality, rapid postpartum return to estrous and selection signatures around genes regulating follicular recruitment and oocyte maturation, whereas Finnsheep show ovulation rates of 3–5 ova per cycle and average litter sizes of 2.5–3.5, with strong responses to flushing and gonadotropins and genomic enrichment for variants in follicle-activation (Hippo) and energy-sensing (AMPK) pathways [79]. Relative to these global high-prolificacy models, Duolang expresses moderate fecundity but valuable growth, carcass and adaptation traits, suggesting that its optimal breeding trajectory lies in incremental, balanced increases in litter size and lambs weaned rather than attempting to emulate the extreme prolificacy of Hu, STH, Romanov or Finnsheep, which would likely exceed the nutritional and management capacity of many southern Xinjiang production systems.

## 11. Environmental and Stress Factors

**Figure 5:**
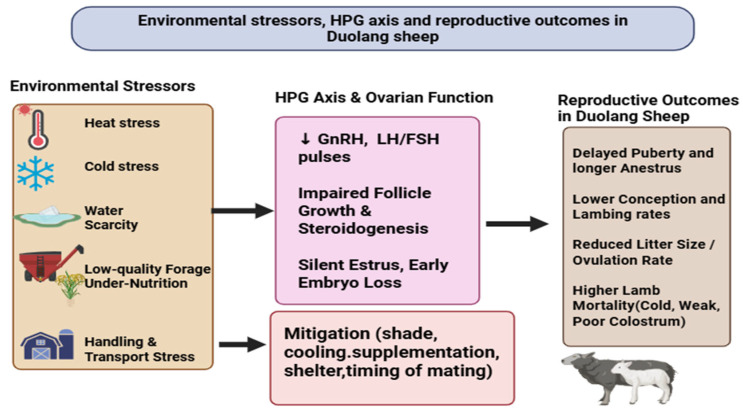
Environmental stressors and reproductive performance in Duolang sheep. The figure shows how environmental stress may disrupt HPG axis activity and ovarian function. These effects may reduce fertility, litter size, and lamb survival. Key mitigation measures include shade, cooling, supplementation, shelter, and proper mating timing.

Environmental stressors, especially temperature extremes, water and forage scarcity, and handling or transport, play a decisive role in shaping reproductive outcomes of Duolang sheep in the arid and semi-arid systems of southern Xinjiang. Heat stress disrupts the hypothalamic–pituitary–gonadal axis, suppresses estrous behavior and conception processes; elevated temperatures increase oxidative stress, damage granulosa cells and depress estradiol synthesis, leading to shortened or silent estrous, lower ovulation rate and greater early embryonic loss. At the same time, in rams, it impairs semen quality, increases sperm DNA fragmentation, and reduces libido [81]. Conversely, severe winter cold diverts energy toward thermoregulation, increases maintenance requirements, lowers body condition score, and indirectly depresses ovulation and conception via prolonged negative energy balance [82]. Peripartum cold exposure increases the risk of neonatal hypothermia, mismothering, and lamb mortality unless shelter, bedding, and wind protection are provided. Seasonal water and pasture shortages further constrain reproduction: even short-term water deprivation reduces intake, alters rumen fermentation, and elevates cortisol, negatively affecting follicular activity and estrous expression, while chronic undernutrition suppresses insulin, leptin, and IGF-1, key metabolic cues for GnRH and LH secretion, resulting in anovulation, weak estrous, and prolonged anestrous [83]. Low-quality, fibrous forage maintains ewes in negative energy balance, delaying puberty, lengthening postpartum anestrous, and reducing ovulation rate; twin and triplet pregnancies are particularly vulnerable, with mid-to-late gestation nutrient deficits predisposing to fetal growth restriction, low birth weight, and elevated perinatal mortality. Handling, transport, gathering, shearing, and flock mixing can trigger acute stress responses marked by high cortisol and catecholamines that suppress reproductive function and reduce conception when they coincide with estrous or early pregnancy; transport during the first trimester has been linked to increased embryo loss and longer inter-lambing intervals. Long-distance transhumant movements typical of Xinjiang add cumulative fatigue, weather exposure, and nutritional fluctuation, further contributing to variable estrous expression and lamb survival in Duolang flocks. Mitigation strategies thus form a critical component of reproductive management. The relationships between environmental stressors, HPG axis function, and reproductive outcomes in Duolang sheep are summarized in Figure 5. The provision of natural or artificial shade, and in semi-intensive systems, sprinklers or evaporative cooling, reduces heat load and improves reproductive outcomes. Continuous access to clean water, energy, and protein-rich supplements, rumen-bypass fats, and improved forage conservation (hay, silage, haylage) helps maintain BCS during mating and late gestation [84]. Ration balancing with adequate selenium, iodine, zinc, and vitamin E enhances ovarian responsiveness, embryo survival, and colostrum quality, supporting lamb viability in cold conditions [85]. In addition, scheduling mating to avoid periods of extreme heat or severe cold, typically targeting early autumn when temperatures are moderate and forage quality improves, reduces thermal and nutritional stress at conception, while the use of hormonal synchronization and the ram effect allow breeding windows to be shifted and tightened away from climatic extremes. Finally, minimizing transport and major handling around estrous and implantation, practicing low-stress weaning, and maintaining calm, predictable herding routines and well-designed handling facilities are essential to limit acute stress episodes and their negative reproductive consequences in Duolang sheep.

## 12. Breeding Programs and Economics

Improving reproductive efficiency in Duolang sheep requires breeding and management strategies that jointly target earlier puberty, a moderate increase in twinning rate, and high lamb survival, the traits with the greatest impact on flock-level productivity in arid-region systems [3]. Evidence from both Chinese and international sheep programs shows that reproductive traits exert a disproportionately large effect on enterprise profitability compared with growth or carcass traits, so breeding goals, selection schemes, and economic evaluation must be explicitly aligned if Duolang sheep are to reach their full potential under Xinjiang conditions [86].

**Figure 6:**
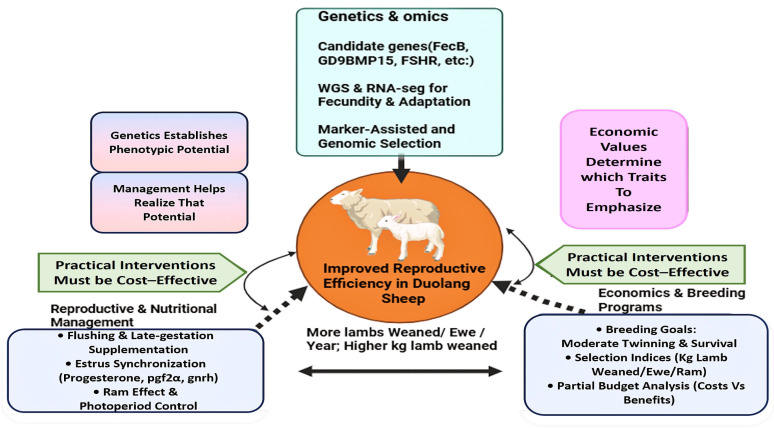
Integrated framework for improving reproductive efficiency in Duolang sheep. The figure summarizes how genetics, management, and economic priorities interact to improve reproductive performance. It highlights candidate genes and genomic tools together with practical reproductive and nutritional interventions. The framework also emphasizes that breeding decisions should remain cost-effective and aligned with production goals.

From a biological standpoint, three core breeding goals are central: early but well-supported puberty, moderate increases in litter size, and robust lamb survival. Age at puberty determines the onset of lifetime reproductive output, and earlier puberty shortens the generation interval and increases the number of lamb crops per ewe, although selection for early puberty must be balanced against adequate body size to avoid dystocia and poor maternal performance. For Duolang, which currently averages ≈1.0 lamb per lambing, a realistic objective is to increase litter size into the 1.4–1.6 range by favoring twinning rather than extreme prolificacy, while simultaneously strengthening perinatal care and ewe nutrition. Because lamb survival to weaning has substantial economic value and moderate heritability, emphasis on maternal behavior, udder health, colostrum yield, and lamb vigor is needed to offset potential increases in mortality associated with higher litter size, especially under the cold stress and forage limitation common in southern Xinjiang [87]. At the genetic-program level, both pure breeding and structured crossbreeding are relevant for Duolang sheep, but they serve different functions. Pure breeding remains the primary route for within-breed improvement while preserving Duolang’s local adaptation and cultural value; here, phenotypic selection on fertility, litter size, lamb survival, growth, and body condition can be integrated with marker-assisted and genomic selection approaches to improve the accuracy of breeding values for low-heritability traits. Given that litter size heritability is typically low (h2 ≈ 0.05–0.15), whereas ovulation rate and growth traits are more heritable, Duolang breeding objectives should be balanced so that gains in prolificacy do not erode adaptation or lamb survival [88]. Crossbreeding, in contrast, can be used strategically to introduce prolificacy or growth attributes from complementary breeds. Maternal crossbreeding with Hu or Small-Tail Han rams may elevate litter size and maternal capacity, but such gains can be negated by higher lamb mortality if feeding and management are not upgraded in parallel. Terminal crossbreeding, in which Duolang ewes are mated to specialized meat sires with all progeny marketed, can improve carcass weight and growth without altering the Duolang maternal base, but any crossbreeding scheme must safeguard environmental resilience, disease tolerance, and the genetic identity of the Duolang breed in Xinjiang systems. The integrated relationships between genetics, management interventions, and economic outcomes in Duolang sheep are summarized in Figure 6.

Economic selection indices and enterprise-level metrics provide the link between genetic decisions and farm profitability. For Duolang sheep, indices should combine reproductive traits (litter size by parity, lamb survival, ewe fertility), growth and carcass traits (weaning weight, post-weaning gain, carcass yield), and adaptive traits (ewe longevity, resistance to climatic and nutritional stress), reflecting the dual emphasis on productivity and robustness in arid environments. Bio-economic modeling consistently shows that the number of lambs weaned per ewe joined and the derived indicator kilograms of lamb weaned per ewe per year (kg./E) carry the highest positive economic weights, because they integrate fertility, prolificacy, and lamb growth into a single profitability driver [89]. Partial-budget analyses suggest that increasing lambs weaned per ewe per year from ≈1.0 to 1.4–1.5 can raise enterprise profitability by about 15–30%. However, these gains may be offset if higher prolificacy leads to increased lamb mortality, greater feed requirements in late gestation and lactation, or higher labor and veterinary costs. For Duolang under Xinjiang conditions, realistic economic comparisons should contrast a baseline scenario of singleton lambings, extensive grazing, minimal supplementation, and limited reproductive technology, with an improved scenario that combines moderate twinning, targeted supplementation, limited but strategic use of synchronization/AI in nucleus flocks, and enhanced lamb survival. In practice, breeding programs should prioritize kilograms of lamb weaned per ewe per year rather than litter size alone, use balanced indices incorporating reproductive, growth and adaptive traits, apply reproductive technologies initially in nucleus and multiplier flocks where management capacity and genetic leverage are greatest, and conduct regular partial-budget evaluations to ensure that genetic gains in prolificacy translate into sustainable net economic benefit rather than hidden increases in costs or losses.

## 13. Future Research Directions

Future work on Duolang sheep reproduction should prioritize integrated, data-rich approaches that link genetics, management, and environment. First, there is an urgent need for standardized longitudinal recording of reproductive, health, and environmental data (e.g., puberty age, mating dates, and estrous expression, litter size by parity, lamb survival, BCS, climate, and feed status) across representative Duolang flocks [75]. Robust electronic flock-recording schemes underpinned by BLUP or single-step genomic evaluations have been pivotal for genetic gain in other sheep industries and would provide the foundation for accurate genetic parameter estimation and selection indices in Duolang. Second, randomized controlled trials (RCTs) of out-of-season breeding strategies are needed under Xinjiang conditions to compare photoperiod manipulation, progesterone-eCG protocols, ram effect-based schemes, and their combinations in terms of conception, lambing distribution, lamb survival, and cost benefit, rather than extrapolating from other breeds and regions [90,91]. Third, the growing availability of Duolang-specific genomic resources, including whole-genome resequencing of 297 Duolang sheep with litter size records, should be exploited to validate candidate markers for fecundity and to implement pilot genomic selection schemes that jointly target litter size, lamb survival, and adaptation. These pilots should test different reference-population designs (nucleus vs. multi-tier recording) and quantify the realized genetic and economic gains relative to conventional selection [75,92].

Finally, sensor and AI-based estrous and health monitoring represent a promising frontier; accelerometer, GPS, and thermal sensors combined with machine-learning algorithms have already been shown to classify small-ruminant behaviors and detect estrous automatically in other systems, and could be adapted for extensive Duolang flocks to improve heat detection, optimize ram introduction, and monitor peripartum risk in real time [93]. Future research should also position Duolang sheep within a broader international framework by comparing them with native and adapted breeds managed under arid, semi-arid, Mediterranean, and extensive pasture-based systems in Europe, North America, South America, Australia, and New Zealand. In Mediterranean Europe, reproductive seasonality has been studied in local breeds such as Rasa Aragonesa, where photoperiod and genotype strongly influence ovulatory activity and breeding season length, and genomic studies have identified loci associated with seasonal reproductive performance [94,95]. In Australia and New Zealand, breeding ewe lambs at 7–9 months has been investigated as a means of improving lifetime productivity, although success depends strongly on liveweight, body condition, and pasture availability [96]. Across international sheep systems, reproductive biotechnologies such as ram effect management, progesterone-based synchronization, prostaglandin protocols, GnRH-based timed breeding, and, in more intensive flocks, photoperiod control are used to concentrate lambing, facilitate out-of-season breeding, and improve labor efficiency [97]. In the Brazilian semi-arid region, comparative studies of native and exotic or crossbred sheep likewise show that reproductive and productive efficiency depends on balancing improved growth potential with adaptation, maternal behavior, and survival under climatic stress [98]. Accordingly, future Duolang studies should quantify not only litter size, but also estrous response, conception rate, lamb survival, lambs weaned per ewe, kilograms of lamb weaned per ewe per year, and the cost effectiveness of each intervention, so that outcomes under Xinjiang conditions can be compared directly with those reported internationally. Together, these research directions would move Duolang reproduction from descriptive studies toward a predictive, precision breeding and management framework, aligning with international trends in genomic and precision livestock breeding while remaining grounded in the ecological and socio-economic realities of southern Xinjiang [99].

## 14. Conclusions

Duolang sheep, an indigenous breed from southern Xinjiang, exhibit significant potential for improvement in reproductive efficiency through a combination of genetic, environmental, and management interventions. Despite their adaptation to the arid and semi-arid conditions of the region, current reproductive performance, characterized by low litter size and relatively high lamb survival, highlights the gap between their genetic potential and actual output. Advances in genomics, particularly the identification of fecundity-related genes such as BMPR1B (FecB), GDF9, and BMP15, provide a robust foundation for enhancing reproductive traits through marker-assisted and genomic selection. However, achieving optimal reproductive efficiency requires integrated management strategies, including nutritional supplementation, hormonal synchronization, and strategic crossbreeding, to address environmental stressors such as heat, cold, and forage scarcity. The development of Duolang-specific genomic selection indices, tailored to the unique production systems of Xinjiang, could facilitate the acceleration of reproductive progress while maintaining the breed’s environmental resilience and meat production capacity. Future research should focus on refining genetic models, conducting controlled breeding trials, and leveraging advanced technologies such as AI-based estrous monitoring to create a predictive, precision-based breeding framework that aligns with the socio-economic and ecological realities of the region.

## Figures and Tables

**Table 1:** Core reproductive performance indicators in Duolang sheep under southern Xinjiang production systems.

Trait	Breed	Typical Value/Range	References
Lambs weaned per ewe per year	Duolang	≈0.8–1.1	[40]
Inter-lambing interval (ILI)	Duolang	≈12 months (lambing occurs annually)	[40]
Lamb survival to weaning (%)	Duolang	≈85–95% (all lambs)	[41,45]
Litter size (lambs per ewe per lambing)	Duolang	≈1.0	[44]
Twinning rate (%)	Duolang	Low; generally <20%	[44,46]
Gestation length (days)	Duolang	≈147–152	[47]

**Table 2:** Summarizes the main reproductive protocols discussed in relation to seasonal breeding management in Duolang sheep.

Protocol	Main Purpose	Main Components	Best Use Context	Key Limitation
Ram effect	To stimulate and synchronize estrous	Introduction of isolated, sexually active rams [56,59,60]	Best near the start of the breeding season [49,56,60]	Less precise; depends on ewe condition [59,60]
Progesterone device + eCG (±PGF_2_α)	To synchronize estrous and support out-of-season breeding	Intravaginal progesterone device/pessary; eCG at removal; PGF2α added in some protocols depending on cyclic status and the presence of functional corpora lutea [57,58,61,62,63]	Useful for advancing breeding activity and FTAI [57,58,61,62,63]	More costly and labor-intensive [51,61,62,63]
PGF_2_α protocol	To synchronize estrous in cycling ewes	Usually, two PGF2α injections 9–11 d apart [64]	Best during the natural breeding season [64]	Ineffective in deep anestrus [64]
GnRH-based protocol	To control ovulation timing	GnRH–PGF2α–GnRH, sometimes with progesterone support [57,58,65]	Useful for FTAI in semi-intensive flocks [65]	More complex in low-input systems [51,65]
Photoperiod manipulation	To advance or extend breeding activity	Artificial light management in housed systems [55,66]	Useful in semi-intensive or elite flocks [55,66]	Requires housing and light control [51,55,66]

**Table 3:** Comparative litter size at birth in Duolang and selected prolific sheep breeds.

Breed	Typical Value/Range (Lambs per Lambing)	References
Duolang	≈1.0	[43,44]
Hu sheep	2.0–2.4	[76,77]
Tan sheep	1.4–1.6	[78]
Romanov	2.7–3.0	[79]
Finnsheep	2.5–3.0	[79]
Small-Tail Han	2.4–2.8	[80]

## Data Availability

No new data were created or analyzed in this study.

## Abbreviations

AI	Artificial insemination
BCS	Body condition score
BLUP	Best linear unbiased prediction
BMPR1B	Bone morphogenetic protein receptor type 1B
BMP15	Bone morphogenetic protein 15
CC BY	Creative commons attribution
CIDR	Controlled internal drug release
DOI	Digital object identifier
E2	Estradiol
Ecg	Equine chorionic gonadotropin
ERα	Estrogen receptor alpha
Erβ	Estrogen receptor beta
FABM	Feed agribusiness management
FecB	Booroola fecundity mutation
FSH	Follicle-stimulating hormone
FSHR	Follicle-stimulating hormone receptor
FTAI	Fixed-time artificial insemination
GDF9	Growth differentiation factor 9
GnRH	Gonadotropin-releasing hormone
GPS	Global positioning system
HPG axis	Hypothalamic–pituitary–gonadal axis
HPO axis	Hypothalamic–pituitary–ovarian axis
h^2^	Heritability
IGF-1	Insulin-like growth factor 1
IU	International units
kg./E	Kilograms of lamb weaned per ewe per year
LH	Luteinizing hormone
lncRNA	Long non-coding RNA
PGF2α	Prostaglandin F2 alpha
PI3K-Akt	Phosphoinositide 3-kinase-protein kinase B
RNA-seq	RNA sequencing
SNP	Single nucleotide polymorphism
STAR	Steroidogenic acute regulatory protein
TGF-β	Transforming growth factor beta
WGS	Whole-genome sequencing
